# Beyond presence and frequency: a determinant-level framework for interpreting AMR dissemination in One Health surveillance

**DOI:** 10.3389/fbinf.2026.1836435

**Published:** 2026-07-03

**Authors:** Varunkumar Asediya

**Affiliations:** 1 Faculty of Biological and Veterinary Sciences, Institute of Veterinary Medicine, Nicolaus Copernicus University in Toruń, Toruń, Poland; 2 Institute of Advanced Studies, Nicolaus Copernicus University in Toruń, Toruń, Poland

**Keywords:** antimicrobial resistance (AMR), decision support, genomic surveillance, mobile genetic elements, one health, resistance determinants, sampling denominators

## Abstract

Genomic sequencing is now routine in One Health antimicrobial resistance (AMR) surveillance, but reports often stop at presence and frequency and do not distinguish a determinant confined to one genomic background from one recurring across lineages, reservoirs, and reporting periods—patterns that warrant different action. We propose the Determinant Dissemination State (Dg-state), a determinant-level interpretation layer that classifies each resistance determinant as Contained, Emerging, Disseminating, or Abstain. We specify the complete decision rule under a single notation: six bounded evidentiary domains; a composite dissemination score with default weights; an uncertainty-adjusted score that discounts weak genomic context and thin sampling; an explicit sampling-adequacy gate; a two-stage classification separating confined recurrence from genuine cross-reservoir or cross-lineage spread; and a classification-stability measure. As a proof-of-concept, and with an accompanying reference implementation, we apply the framework to a literature-anchored simulated surveillance dataset with known ground truth, where it reproduces the intended labels and behaves robustly. Dg-state does not infer transmission; it provides an auditable, reproducible way to prioritise determinants for escalation, targeted resampling, and higher-resolution genomic investigation.

## Introduction

Whole-genome sequencing has become a routine component of antimicrobial resistance (AMR) surveillance across veterinary, food-chain, and environmental systems, generating determinant calls, lineage groupings, and metadata spanning livestock, farm environments, slaughter settings, food products, and wastewater (Baker et al., 2023; Argimón et al., 2020; Kayama et al., 2023; Djordjevic et al., 2024). Capability is advancing quickly, yet routine reporting often stops at organism- or compartment-level presence and frequency, without a determinant-level view of dissemination (Centner et al., 2026).

Not all recurrence signals carry the same implication. Repeated detection within one genomic background can reflect lineage persistence, whereas detection across backgrounds, reservoirs, or reporting periods is more consistent with broader distribution and may warrant escalation (Wyres and Holt, 2016). Reports frequently conflate these patterns even though they call for different follow-up. The distinction is interpretative, not mechanistic: distribution across lineages or reservoirs can reflect dissemination, repeated introduction, or sampling structure (Didelot et al., 2017).

Two constraints complicate interpretation. First, genetic context is often only partly resolved: short reads detect determinants but frequently cannot resolve whether they sit on the chromosome or on a plasmid or other mobile element, while long-read and hybrid approaches that resolve context are not consistently available in routine programmes (Ko et al., 2022; Peng et al., 2025; Partridge et al., 2018). Second, recurrence depends on sampling: apparent rarity can reflect sparse coverage or shifting denominators, and non-detection under weak sampling is not evidence of absence (Walsh, 2018; Van Boeckel et al., 2019). Interpretation should therefore treat contextual resolution as graded evidence and make sampling support explicit.

We propose the Determinant Dissemination State (Dg-state), a determinant-level interpretation layer that can be reported routinely and that makes its evidentiary limits explicit. The resistance determinant—an AMR gene, resolvable variant, or resistance-conferring mutation—is the most stable analytical unit, typically available even when genetic structure is unresolved; where context is resolved, the reporting unit can be refined to a determinant-unit (Boolchandani et al., 2019). Dg-state moves beyond detected/not-detected by assigning decision-oriented states, and we give the full rule and a worked, reproducible proof-of-concept so that it can be evaluated and implemented rather than only described.

## The Dg-state framework

### Evidentiary domains

For each determinant *d* we compute six bounded metrics (each in [0, 1]) directly from surveillance data; these are observed signals, not estimates of unobserved parameters. Contextual resolution is treated as evidence *quality* rather than dissemination *magnitude*, and so enters the uncertainty-adjusted score rather than the spread score.

The per-stratum sampling adequacy turns denominator support into a bounded weight, where *n*_r,t_ is the number of units examined and *n* ^*^_r,t_ the programme target:

$$a_{r,t}=\min\left(\;1,n_{r,t}\;/\;n_{r,t}^{*}\;\right)$$

Each detection carries a contextual-resolution class *ρ* ∈ {0,1,2,3}: R0 (detected without isolate-resolved context), R1 (detected in an isolate assembly without a resolved determinant-unit), R2 (partial contextual indicators such as plasmid replicons or integrase/transposase markers), and R3 (determinant-unit resolved by hybrid or long-read assembly). The context score is the detection-weighted mean class, normalised to [0, 1], over the *D*_d_ detection events of *d*:

$$X_{d}=\left(\;1\;/\;D_{d}\;\right)\;\cdot \;\Sigma_{e}\;\rho_{e}\;/\;3$$

With *k*_d,r,t_ detections among *n*_r,t_ examined units, the observed detection proportion is *p*_d,r,t_ = *k*_d,r,t_/ *n*_r,t_. Burden and occupancy are then adequacy-weighted means across all strata Ω, down-weighting poorly supported strata (𝟙?[·] is the indicator):

$$Q_{d}=\left(\;\Sigma_{\Omega }\;a_{r,t}p_{d,r,t}\;\right)\;/\;\left(\;\Sigma_{\Omega }\;a_{r,t}\;\right)\;O_{d}=\left(\;\Sigma_{\Omega }\;a_{r,t}\;1\left[k_{d,r,t}\;>\;0\right]\right)\;/\;\left(\;\Sigma_{\Omega }\;a_{r,t}\;\right)$$

Breadth is computed over supported detections — those in strata with adequacy at or above a floor — so it is not inflated by undersampled strata:

$$\begin{array}{ll} V_{d} & =\left(\;\left|\mathrm{Res}\left(d\right)\right|‐\;1\;\right)\;/\;\left(\;R\;‐\;1\;\right)\;T_{d}=\left(\;\left|\mathrm{Tint}\left(d\right)\right|‐\;1\;\right)\;/\;\left(\;W\;‐\;1\;\right)\;B_{d}\\ & =\left(\;\left|\mathrm{Lin}\left(d\right)\right|‐\;1\;\right)\;/\;\left(\;G\;‐\;1\;\right) \end{array}$$

Each breadth term is 0 when its denominator would be 0. Occupancy and burden together capture breadth-of-presence and detection level; a recurrence-intensity term used in earlier formulations is redundant with these under adequacy weighting and is omitted, leaving a set of non-redundant domains. These metrics summarise distribution and do not, on their own, imply any transmission mechanism (Didelot et al., 2017; Watt et al., 2025).

### Composite score, adequacy gate, and classification

The composite dissemination score is a weighted mean of the magnitude domains, with non-negative weights summing to one (context is excluded here; it enters the uncertainty-adjusted score below):

$$S_{d}=w_{V}V_{d}+w_{B}B_{d}+w_{T}T_{d}+w_{O}O_{d}+w_{Q}Q_{d}$$

Default weights give cross-reservoir and cross-lineage breadth the most mass (*w*_V_ = *w*_B_ = 0.25, with *w*_T_ = 0.15, *w*_O_ = 0.20, *w*_Q_ = 0.15), so that the breadth-and-persistence axis (V, B, T) carries 0.65, because the Contained-versus-Disseminating contrast is fundamentally one of breadth. A high score resting on weak evidence is then discounted: with ā_d_ the mean adequacy over occupied strata, an evidence-quality factor combines context and adequacy, and the uncertainty-adjusted score shrinks *S*_d_ toward caution without zeroing a genuine spread signal:

$$E_{d}=\omega_{X}X_{d}+\omega_{a}\;{ā}_{d}\;{Ŝ}_{d}=S_{d}\;\cdot \;\left[\;\gamma +\left(1\;‐\;\gamma \right)\;E_{d}\;\right]$$

with defaults *ω*_X_ = *ω*_a_ = 0.5 and *γ* = 0.5. Classification proceeds only if the adequacy gate *A*_d_ passes, which requires all of the following to hold; otherwise the determinant is assigned Abstain.

- Summed adequacy over strata with a detection is at least *A*_min_;
- At least *m*_min_ (≥ 2) strata have adequacy at or above the floor and a detection, so no single stratum can drive a call;
- Lineage is assigned for at least a fraction *λ*_min_ of positive isolates; and the determinant calls are valid.

These constants are calibration parameters; the proof-of-concept used an adequacy floor of 0.5, *A*_min_ = 1, *m*_min_ = 2, and *λ*_min_ = 0.8.

A single threshold on a composite score cannot separate Contained from Emerging, because Contained reflects high recurrence within a narrow spread, whereas Emerging reflects spread that is only beginning. The rule is therefore two-stage—gate, then breadth, then magnitude.

if A_d = 0: → Abstain
elif |Lin(d)| ≤ 1 and |Res(d)| ≤ 1: → Contained
elif Ŝ_d ≥ τ_2_: → Disseminating
elif Ŝ_d ≥ τ_1_: → Emerging
else: → Contained (marginal spread)

with 0 ≤ τ_1_ < τ_2_ ≤ 1; a determinant confined to one reservoir and one lineage is Contained regardless of how persistent or abundant it is. The stability of a threshold-mediated label is its distance to the nearest boundary, normalised by the band width (high = robust); a more rigorous alternative is the fraction of weight perturbations that preserve the label:

$$\Phi_{d}=\min\left(\;1,\min\left(\;\left|{Ŝ}_{d}\;‐\;\tau_{1}\right|,\left|{Ŝ}_{d}\;‐\;\tau_{2}\right|\right)\;/\;\left(\;½\left(\tau_{2}\;‐\;\tau_{1}\right)\;\right)\;\right)$$

### Implementation and calibration

**FIGURE 1:**
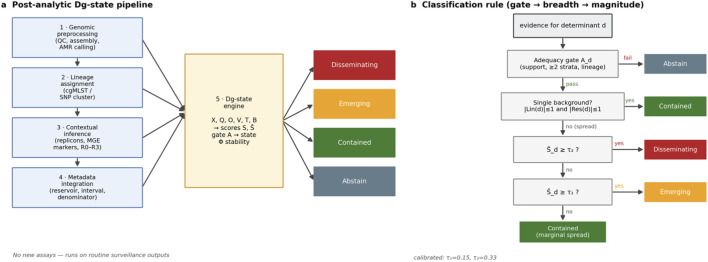
**(a)** The post-analytic Dg-state pipeline: routine genomic, lineage, context, and metadata outputs feed a determinant-level engine that emits one of four states; no new assays are required. **(b)** The classification rule—an adequacy gate (failure → Abstain), a single-background test (→ Contained), then magnitude thresholds on the uncertainty-adjusted score Ŝ separating Emerging from Disseminating. Thresholds shown are the calibrated values used in the proof-of-concept.

Dg-state is a post-analytic layer requiring no new assays beyond a programme’s usual pipeline. Samples are sequenced and determinants called against curated databases; isolates are assigned to lineage groups (e.g., cgMLST complexes or SNP clusters); each detection is given a contextual class R0–R3 from local features and, where available, long-read assemblies; and calls are linked to reservoir, interval, and denominator. The engine then computes the domains, the composite and uncertainty-adjusted scores, the gate, the state, and the stability (Figure 1). A minimal implementation needs four harmonised tables—determinant calls, lineage assignments, reservoir–time metadata, and sampling denominators (Sherry et al., 2023; Hawkey et al., 2021; Ikhimiukor et al., 2024; Macesic et al., 2023; Munk et al., 2022).

Weights and thresholds are intended to be calibrated retrospectively against adjudicated outcomes, via a regularised objective trading the mean lead-time gain *ΔL* against the false-escalation rate *F* and the abstention rate *ψ* while regularising toward prior weights *w*_0_:

$$\max_{w,\tau 1,\tau 2}\alpha \Delta L\;‐\;\beta F\;‐\;\delta \psi \;‐\;\eta \;{\left\|w\;‐\;w_{0}\right\|}_{2}^{2},\Delta L=\theta_{\mathrm{std}}\;‐\;\theta_{\mathrm{Dg}},F=N_{\mathrm{false}}/N_{\mathrm{esc}},\psi =N_{\mathrm{abs}}/N_{\mathrm{eval}}$$

subject to *w* ≥ 0, Σ*w* = 1, and 0 ≤ τ_1_ < τ_2_ ≤ 1, with non-negative weights α, β, δ and regularisation strength η. These quantities should be read jointly: lead-time gains are uninformative if achieved through excessive false escalation or indiscriminate abstention.

### Proof-of-concept

Dg-state is denominator-aware, and public genomic repositories—including the NCBI Pathogen Detection Isolates Browser—record detections but not the per-stratum sampling denominators that Dg-state requires, which reside in programme sampling frames. A faithful demonstration therefore cannot be drawn from public sequence data alone, so we use a controlled simulation with known operational ground truth, parameterised to published patterns, against which the engine’s labelling can be checked directly. We frame this as a proof-of-concept and internal-consistency check, not external validation.

The dataset comprises five reservoir classes (poultry, cattle, slaughterhouse environment, retail meat, wastewater), eight quarterly intervals (forty reservoir–time strata in total), and up to fourteen lineage groups, with heterogeneous denominators against a target of sixty units. Sixteen determinants, four per state, instantiate the four operational categories, anchored to published figures: a broadly distributed, plasmid-resolved archetype (*blaNDM*-like; Acman et al., 2022); an early cross-reservoir, short-read-only archetype (*mcr-1*-like) at within-reservoir proportions ranging from a few percent to about 25%, as reported for *mcr-1* in livestock; and clonally restricted archetypes confined to one lineage and reservoir. Each determinant’s generative label served as the known ground truth.

At thresholds calibrated against the known labels (τ_1_ = 0.15, τ_2_ = 0.33) the engine reproduced the intended operational label for all sixteen determinants (Table 1), confirming that the specification is internally consistent and implementable rather than demonstrating predictive accuracy. The behaviour also differed from conventional reporting in the way most relevant to surveillance: a frequency-only ranking prioritised locally abundant but confined determinants over the emerging cross-reservoir signal, whereas Dg-state separated them through the breadth gate and spread-weighted score. Classifications were robust—mean label preservation was 0.99 under weight perturbation, and the intended labels were recovered across a broad range of thresholds rather than at a knife-edge. A reference implementation reproduces Table 1 and can be applied unchanged to programme data; prospective evaluation within a programme that records its sampling frame—comparing Dg-state escalation against adjudicated follow-up—is the necessary next step and the appropriate test of decision utility.

**TABLE 1:** Determinant-level reporting structure on the proof-of-concept dataset.

Determinant	Ctx	Res	Lin	O	Ŝ	Dg-state	Stab	Surveillance action
*blaNDM*	R2	5	7	0.87	0.608	Disseminating	1.00	Escalate; long-read/ MGE follow-up
*tetX4*	R2	5	5	0.77	0.528	Disseminating	1.00	Escalate; long-read/ MGE follow-up
*blaKPC*	R2	4	6	0.77	0.516	Disseminating	1.00	Escalate; long-read/ MGE follow-up
*aac(6′)-Ib*	R2	4	5	0.73	0.489	Disseminating	1.00	Escalate; long-read/ MGE follow-up
*blaOXA-48*	R1	3	3	0.37	0.267	Emerging	1.00	Targeted resampling; context confirmation
*ermB*	R1	3	3	0.37	0.249	Emerging	1.00	Targeted resampling; context confirmation
*mcr-1*	R1	2	3	0.26	0.201	Emerging	1.00	Targeted resampling; context confirmation
*qnrS*	R1	2	2	0.25	0.171	Emerging	0.92	Targeted resampling; context confirmation
*blaCMY* (clonal)	R1	1	1	0.21	0.126	Contained	1.00	Routine monitoring
clonal-Hi	R1	1	1	0.19	0.121	Contained	1.00	Routine monitoring
*sul1* (clonal)	R1	1	1	0.18	0.116	Contained	1.00	Routine monitoring
*dfrA1* (clonal)	R1	1	1	0.16	0.097	Contained	1.00	Routine monitoring
rareB	R0	1	0	0.03	0.005	Abstain	1.00	Insufficient evidence; improve sampling
envX1	R0	0	0	0.00	0.001	Abstain	1.00	Insufficient evidence; improve sampling

A representative subset of the sixteen determinants is shown (two Abstain rows omitted for space). Stability is the label-preservation fraction under weight perturbation. The single-background gate makes Contained independent of detection level: clonal-Hi, despite frequent detection, is confined to one reservoir and one lineage and stays Contained, whereas the more broadly distributed mcr-1 (two reservoirs, three lineages) is Emerging—a distinction frequency-based reporting does not make. Ctx, representative contextual class; Res, reservoirs; Lin, lineage groups; O, occupancy.

## Discussion

Programmes already call determinants, assign genomic backgrounds, and sometimes add context; what is often missing is a determinant-level statement of distribution—whether a signal is confined to one background or recurring across backgrounds, reservoirs, and reporting intervals in a way that should trigger follow-up (Kayama et al., 2023; Ikhimiukor et al., 2024; Centner et al., 2026; Muloi et al., 2023). Dg-state supplies that statement as an explicit, auditable interpretation layer.

The state labels are meant to guide action. Emerging should prompt targeted resampling, better context, and tracking across reservoirs; Disseminating supports escalation and deeper follow-up, including long-read sequencing where informative for mobile-element context; and Abstain is a valid outcome that reports too-weak sampling or context rather than forcing a call (Baker et al., 2023; Djordjevic et al., 2024; Ferdinand et al., 2021; Grad and Lipsitch, 2014). In One Health systems, early warning often appears as low-level recurrence across livestock, slaughter environments, food-chain interfaces, and wastewater, which reservoir-by-reservoir reporting can hide (Hendriksen et al., 2019; Munk et al., 2022; Larsson and Flach, 2022).

Dg-state describes surveillance signals; it does not explain mechanisms. A determinant seen across genomic backgrounds suggests broader distribution but does not prove horizontal gene transfer, and multi-reservoir detection can reflect dissemination, repeated introductions, shared sources, or sampling structure (Allen et al., 2010; Woolhouse et al., 2015; McEwen and Collignon, 2018). It should be read as structured reporting within a defined sampling system, not a transmission or outbreak model.

The main advantage is transparency: the evidentiary domains show why a determinant received a state and what data would change the call; the composite score is a bounded index, not a probability; and treating contextual resolution as evidence quality, separate from spread magnitude, keeps the score interpretable and explains why an unresolved but broadly distributed determinant is escalated cautiously (Emerging) rather than prematurely (Disseminating). The principal limitation is that the demonstration here is a proof-of-concept rather than external validation, which is constrained by the absence of per-stratum denominators in public archives; cross-programme comparison will also require harmonised inputs, and thresholds must be calibrated to local sampling design (Grad and Lipsitch, 2014; Watt et al., 2025). A lightweight path to adoption is to agree reservoir and reporting-interval definitions, a routine lineage grouping, and minimum per-stratum denominators, then pilot Dg-state alongside existing reports as a triage layer and record how escalations align with follow-up.

## Conclusion

Genomic AMR surveillance produces rich data, but routine reporting rarely turns determinant detections into decision-relevant interpretation across reservoirs, time windows, and genomic backgrounds. Dg-state is a lightweight, auditable interpretation layer that separates confined signals from broader recurrence and makes uncertainty explicit through Abstain, specified here as a complete decision rule with a reproducible proof-of-concept. We encourage programmes to pilot determinant-level state reporting alongside current summaries, calibrate thresholds to their sampling design, and share implementations and conventions so that interpretation becomes comparable across One Health systems.

## Data Availability

The data underlying the proof-of-concept were generated by simulation; the full specification used to generate the dataset (evidentiary domains, default weights, and classification thresholds) is provided in the article. The reference implementation that reproduces Table 1 is available from the author upon reasonable request.
